# High Risk of Chronic Endometritis in Isthmocele—A Systematic Review and Meta-Analysis

**DOI:** 10.3390/jcm14113628

**Published:** 2025-05-22

**Authors:** Angela Vidal, Janna Pape, Vithusha Vinayahalingam, Marietta Gulz, Tanya Karrer, Michael von Wolff

**Affiliations:** 1 Division of Gynecological Endocrinology and Reproductive Medicine, Women’s University Hospital, Inselspital Bern, University of Bern, 3010 Bern, Switzerland; janna.pape@insel.ch (J.P.); michael.vonwolff@insel.ch (M.v.W.); 2 Department of Gynecology and Obstetrics, Hospital Wolhusen, 6110 Wolhusen, Switzerland; vithusha19@hotmail.com; 3 Department of Gynecology and Obstetrics, Bern University Hospital, University of Bern, 3010 Bern, Switzerland; marietta.gulz@insel.ch; 4 Medical Library, University Library of Bern, University of Bern, 3012 Bern, Switzerland; tanya.karrer@unibe.ch

**Keywords:** isthmocele, chronic endometritis, caesarean section, inflammation

## Abstract

**Background**: In recent decades, there has been a notable rise in the prevalence of caesarean sections, which has been accompanied by a concomitant increase in the incidence of long-term complications, including the occurrence of isthmocele. This anatomical alteration has been associated with symptoms such as abnormal uterine bleeding (AUB), chronic pelvic pain, and secondary infertility. On the other hand, chronic endometritis (CE), characterised by the infiltration of plasma cells into the endometrium, is also associated with infertility and repeated implantation failures. Given the coincidence of symptoms between these two conditions, the question arises as to whether there is an association between isthmocele and CE. **Objective**: This systematic review and meta-analysis aim to examine the association between isthmocele and CE and to assess its potential clinical implications. **Methods**: A systematic literature search was conducted in Medline, Embase, and Cochrane CENTRAL up to February 2025. The inclusion criteria were studies involving histopathological findings in isthmocele. The results of the meta-analysis incorporated observational studies and trials to evaluate the prevalence of CE in women with isthmocele, as well as the odds ratios (OR) for CE in isthmocele compared to women without isthmocele, and for CE in isthmocele with AUB compared to women without AUB. **Results**: A total of seven studies were included in the systematic review, comprising 976 women in the systematic review and 876 women in the meta-analysis. The pooled prevalence of CE in women with an isthmocele was 40% (95% CI: 24–58%). Here, the risk of CE was threefold higher in the presence of an isthmocele as compared to its absence (OR = 3.01; 95% CI: 1.02–9.03). Furthermore, the risk of CE was further increased in women with AUB and isthmocele compared to those without AUB (OR = 6.33, 95% CI: 1.94–20.67). **Conclusions**: CE and isthmocele are both under-diagnosed and poorly understood conditions. The high prevalence of CE in isthmocele indicates a substantial clinical burden. Specifically, women with AUB and isthmocele exhibit a six-fold higher risk of developing CE. These results underscore the importance of recognising isthmocele as a significant risk factor for CE, particularly in women presenting with isthmocele-associated AUB. Further research is warranted to elucidate the underlying mechanisms and to develop targeted interventions for the prevention and management of CE in this population.

## 1. Introduction

Given the significant increase in caesarean section rates over the past decade, isthmocele has been observed as a common long-term complication associated with subsequent gynecologic comorbidities [1,2,3]. The prevalence of isthmocele in patients after caesarean section has been documented to range from 24% to 80%, with a significant negative impact on global health [3,4,5].

Isthmocele has been associated with a variety of symptoms, including abnormal uterine bleeding (AUB), chronic pelvic pain, and secondary infertility [1,6]. An association between the development of an isthmocele and suturing has been identified as a possible risk factor. Vacon-Marceau et al. (2017) found that the double-layer closure technique could be linked to better integrity of the lower uterine segment in later pregnancies [7]. Di Siezio-Sardo et al. (2017) found no major differences in defects between single-layer and double-layer closure after caesarean section, but noted a thinner residual myometrium in single-layer cases [8]. A study by Alper et al. in 2024 suggests that the parallel layer technique may reduce isthmoceles and increase the thickness of the residual myometrium [9].

A significant impact of isthmocele on reproductive outcomes has been observed, particularly in assisted reproductive technology, where affected women have lower implantation, clinical pregnancy, and live birth rates [10,11,12]. In this regard, several mechanisms have been postulated to explain the contribution of the isthmocele to secondary infertility. Anatomical disruption caused by the scar defect may lead to the accumulation of intrauterine fluid (e.g., mucus or blood), which may impede sperm transport and embryo implantation. Furthermore, fibrosis and impaired healing at the scar site may result in altered uterine contractility [13,14], thereby interfering with the positioning and transfer of the embryo [15,16]. At the molecular level, microbial imbalance, chronic inflammation, and local immune dysfunction within the scarred area may lead to impaired endometrial receptivity and contribute to a hostile environment for implantation [10,12,13].

Chronic endometritis (CE) is defined as a persistent inflammation of the endometrium characterised by the infiltration of plasma cells [17,18]. Although often asymptomatic, CE has been increasingly recognised as a factor contributing to abnormal uterine bleeding, infertility, and recurrent implantation failure [18,19,20]. The definition and diagnostic criteria for CE vary across studies. However, the presence of CD138-positive plasma cells in the endometrial stroma is most commonly used as the primary histological marker [21,22,23].

Although there has been progress in the understanding of the immunological basis of CE, the precise mechanisms by which chronic inflammation alters endometrial receptivity are incompletely characterised, particularly the impact of the isthmocele on the endometrial immune system. Clinical overlap in symptoms such as abnormal uterine bleeding, pelvic pain, and infertility observed in both isthmoceles and CE raises important questions regarding a possible pathophysiological link between the two conditions. Given the rising occurrence of caesarean scar defects and the mounting evidence for the role of CE in reproductive health, it is imperative to explore this association. Nevertheless, only a limited number of studies have systematically investigated this association.

This systematic review and meta-analysis aims to evaluate the association between isthmocele and CE and to assess its potential clinical implications.

## 2. Materials and Methods

### 2.1. Registration of Protocols

The protocol was registered in the Prospective International Registry of Systematic Reviews, PROSPERO (registered number CRD CRD420251011611). The guidelines for the Preferred Reporting Items for Systematic Reviews and Meta-Analyses (PRISMA) have been used [24].

### 2.2. Search Strategy

To identify potentially relevant publications on the topic, two main search concepts were developed: (1) chronic endometritis and (2) isthmocele. A search strategy was designed and investigated in MEDLINE, Embase, CENTRAL, Cochrane Database of Systematic Reviews, and Scopus. In addition, registered trials were searched on clinicaltrials.gov. A medical librarian specialist (T.K.) developed an initial search strategy in Medline and tested it against a list of core references to ensure key publications were included. After refinement, the information specialist set up the search strategy for each information source based on database-specific index terms and free text. The free text search included synonyms, acronyms, and similar terms. The search was finalised on 3 February 2025. The results were deduplicated using the automated deduplication tool of Deduklick [25]. Screening, data extraction, and study assessment was performed in the screening tool Covidence.

### 2.3. Inclusion and Exclusion Criteria

Studies were independently assessed for inclusion using Covidence software (www.covidence.org) [26] by the investigators AV and VV. The eligibility was based on original articles revealing the association between isthmocele and CE. We excluded trials with an inadequate design.

### 2.4. Data Extraction

The extracted data were independently summarised and reviewed by two investigators (AV and VV). Primary variables of interest included study population characteristics such as patient age, number of CS, presence of isthmocele and CE, methods and parameters used to diagnose CE, AUB, secondary infertility, residual myometrial thickness (RMT), presence of endometriosis, and any further interventions. Disagreements were discussed and resolved by consensus. The results are shown in Table 1.

### 2.5. Quality Assessment

The Newcastle–Ottawa scale (NOS) was utilised to evaluate the quality of the individual studies [27]. Three parameters were considered for the individual study scoring: subject selection (0–4 stars); comparability (0–2 stars); and study outcome (0–3 stars). The scoring was composed as follows: good quality (=3 or 4 stars in the selection domain AND 1 or 2 stars in the comparability domain AND 2 or 3 stars in the outcome/exposure domain); fair quality (=2 stars in the selection domain AND 1 or 2 stars in the comparability domain AND 2 or 3 stars in the outcome/exposure domain); and poor quality (=0 or 1 star in the selection domain OR 0 stars in the comparability domain OR 0 or 1 stars in the outcome/exposure domain). All the studies included were reviewed by AV and CB to independently assess the risk of bias. Disagreements were resolved by consensus. Scoring was conducted according to the terms listed in Table 2.

### 2.6. Data Synthesis

The primary outcome investigated the pooled prevalence of CE in women with isthmocele. Further, pooled ORs for CE were calculated in women with and without isthmocele, as well as in women with and without AUB and isthmocele. All the statistical analyses were performed with the “metaphor” function of the R software, version 4.4.3 (R Core Team, Vienna, Austria, 2013). Heterogeneity was examined using Cohen’s Q statistic and the I^2^ statistic. In the presence of high heterogeneity, random-effects models were used.

## 3. Results

### 3.1. Results of the Systematic Review

**Figure 1:**
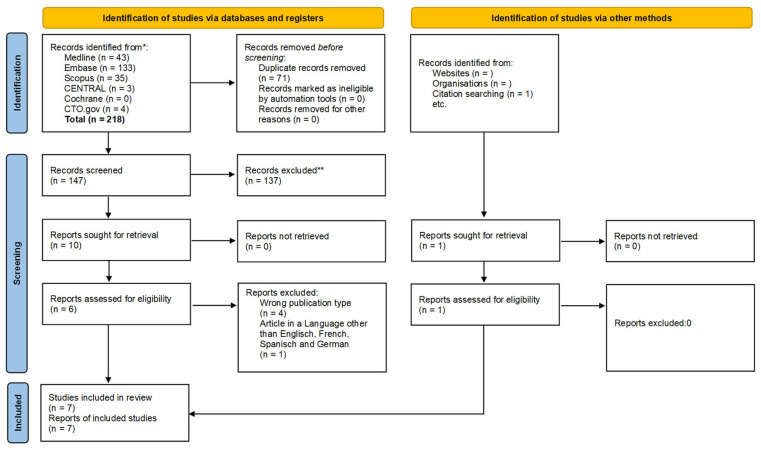
PRISMA flow chart. Flowchart of the bibliography search and selection process. * Consider, if feasible to do so, reporting the number of records identified from each database or register searched (rather than the total number across all databases/registers). ** If automation tools were used, indicate how many records were excluded by a human and how many were excluded by automation tools.

A total of 218 studies were identified by searching the databases. After screening the abstracts and the full text of the study topic, 10 studies remained. However, we excluded 4 of these studies as they did not fit our predetermined inclusion criteria. One study was added manually. Therefore, we included 7 articles in the systematic review (Figure 1).

### 3.2. Study Characteristics

The characteristics of the study populations are summarised in Table 1. Two studies were prospective, and the remaining five studies were retrospective. All the studies were conducted in Asia and were published between 2019 and 2024. A total of 976 women were included in the review. A total of 876 women (89.8%) were eligible for the quantitative analysis. Study sample sizes varied from 16 to 501 patients. The methodological quality of the studies was rated as good for three studies [28,29,30], poor for another three [31,32,33], and one study was rated as fair [34] (Table 2).

**Table 1:** Review of the literature on the association of CE and isthmocele.

First Author, Year of Publication	Study Design	Total Study Population	Number of Participants of Interest (Total)	Number of Participants in Control Group (Total)	Age (y) Mean SD or Median	Age (y) Mean SD or Median (Control Group)	Parameters	Chronic Pain/Pelvic Pain Associated	Infertility Associated (Number/Total)	Endometriosis Associated (Number/Total)	Antibiotic Therapy
Glukhov E. Yu. et al., 2019 [31]	prospective observational incomparable study	50 (CSD)	26/50 (CE)	24/50 (non CE)	33.5	32.0	CE group: 14/26 Non CE group: 6/24	NM	CE group: 15/26 Non CE group: 9/24	CE group: 10/26 Non CE group: 8/24	Yes Most patients received intraoperative intravenous Amoxiclav (92%) or ceftriaxone (8%); in cases of severe CE, antibiotics were continued for up to 5 days.
Higuchi et al., 2022 [32]	retrospective case-control study	84	63 (CSS) Secondary infertility, LSK repair of CSD	21 (non-CSS) Underwent HE, with a history of CS	35.9 ± 3.3	43.6 ± 3.3	CD138, CD3, CD20, CD56, CD 68, CD138, MPO, Tryptase CD138 significantly higher in the CSS group CD138+ plasma cells in 5 HPFs within 2500 μm from cavity side of CSD CD3, CD20, CD68, tryptase was significantly lower in the CSS group	NM	63/63 (Secondary infertility was inclusion criteria)	NM	no
Yang et al., 2021 [33]	case-control study	16	9/16 (CS with CSD)	7/16 (vaginal delivery)	32.2 ± 3.5	33.8 ± 3.1	the MIP-1alpha, SDF-1alpha, IL-27, IL-1beta, IL-2, IL-4, IL-5, IP-10, IL-6, IL-7, IL-8, IL-10, Eotaxin, IL-12p70, IL-13, IL-17A, IL-31, IL-1RA, RANTES, IFN-γ, GM-CSF, TNF-α, MIP-1β, IFN- α, MCP-1, IL-9, TNF-β, GRO-α, IL-1α, IL-23, IL-15, IL-18, IL- 21, and IL-22	NM	16/16	NM	NM
Nobuta et al., 2022 [34]	retrospective case-control study	201	38 (CSS)	163 (Non-CSS)	38	37	CD138, TNF-α, IL-1β ≥1 CD138+ plasma cell per 10 HPFs (random fields) only in endometrial stroma TNF-α (pg/mL): 0.88 (CSS), 0.15 (non-CSS) IL-1β (pg/mL): 17 (CSS), 10 (non-CSS)	NM	201/201	51/73 (only from the CSS group)	No
Wei et al., 2022 [28]	retrospective study using propensitiy score matching	501	170 (CSS)	331	34.9 ± 4.3	34.98 ± 4.9	CD138 CE defined by any of the following: (1) >1 plasma cell per HPF, (2) >5 plasma cells per HPF, (3) >1 plasma cell per section, (4) >1 plasma cell per 10 HPFs, or (5) plasmacyte density index ≥ 0.25.	NM	NM	NM	NM
Wang et al., 2024 [29]	retrospective case-control study	155 with CSD (69 AUB, 86 without AUB)	30 (AUB after PSM)	30 (non AUB after PSM)	37.6 ± 2.6	35.9 ± 4.6	CD138, CD 31 ≥1 CD138+ plasma cell per HPFc	NM	4/15 (AUB) 2/9 (Non-AUB)		Yes not descriptive
Zhang et al., 2024 [30]	retrospective case-control study	64	44 (CSS)	20 (CS with TLH)	30.0 ± 4.3	30.25 ± 4.8	CD138 CE defined as ≥1 CD138+ plasma cell per HPF in 10 HPFs. Mild CE: 1 cell/HPF; Severe CE: ≥2 cells/HPF.	5/44(CSS)	7/44 (CSS)	NM	NM

**Table 2:** Newcastle–Ottawa quality assessment form for cohort studies.

	Selection				Comparability	Outcome				
First Author, Year of Publication	Representativeness of Exposed Cohort	Selection of Non-Exposed Cohort	Ascertainment of Exposure	Outcome of Interest Not Present at Study Start	Comparability of Cohorts on the Basis of the Design or Analysis Controlled for Confounders	Assessment of Outcome	Sufficient Length of Follow-Up for Outcomes to Occur	Adequacy of Follow-Up of Cohorts	Total	Quality Assessment
Glukhov E.Yu. et al., 2019 [31]	★	-	★	-	-	-	★	★	(4/8)	poor
Higuchi et al., 2021 [32]	★	-	★	-	-	-	-	-	(3/8)	poor
Yang et al., 2021 [33]	★	-	-	-	-	-	-	-	(1/8)	poor
Nobuta et al., 2022 [34]	★	-	★	-	★	★	★	★	(6/8)	fair
Wei et al., 2022 [28]	★	★	★	-	★	★	★	★	(7/8)	good
Wang et al., 2024 [29]	★	★	★	★	★	★	-	★	(7/8)	good
Zhang et al., 2024 [30]	★	-	★	★	★	★	-	★	(7/8)	good

There was variation in the diagnostic approach to CE across the studies. Four studies diagnosed CE using immunohistochemical staining for CD138-positive plasma cells, while two studies did not clearly define their diagnostic criteria [29,30]. One of these focused on the association between CD138 and isthmocele, while the other explored the correlations between intrauterine microbiota and inflammatory markers [33]. One study diagnosed CE based on hysteroscopic criteria, with histopathology performed only in unclear cases [31].

Some 5 of the 7 included studies directly examined the presence of CE in women with caesarean scar defects. The reported prevalence of CE in this population ranged from 24% to 79.5%.

**Figure 2:**
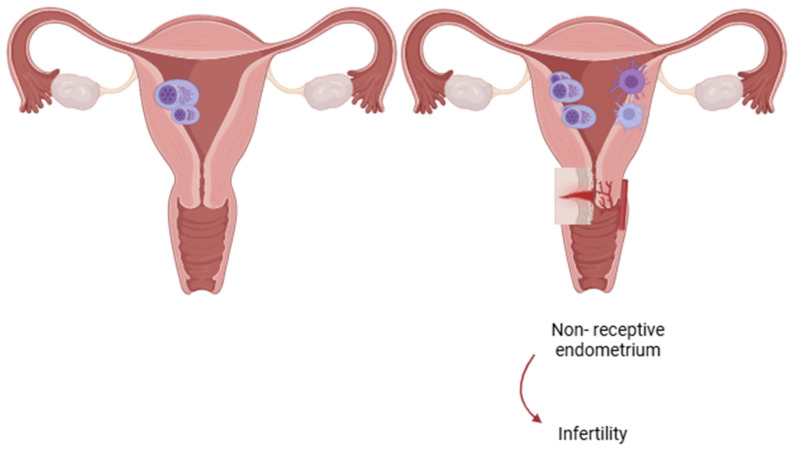
A balanced uterine immune cell environment—involving uNK cells, T cells, macrophages, and dendritic cells—is essential for proper endometrial receptivity. This balance may be disrupted in cases of isthmocele through similar mechanisms. Such imbalances may lead to implantation failure and infertility. Original, created with Biorender.com.

All the studies that utilised immunohistochemical staining found an increased presence of CD138-positive plasma cells in the isthmocele group. These findings support the hypothesis that isthmocele may act as a structural and immunological niche promoting low-grade chronic inflammation in the endometrium (Figure 2).

### 3.3. Results of the Meta-Analysis

A meta-analysis of 5 studies [28,29,30,31,34] involving 876 women was conducted to evaluate the association of chronic endometritis and isthmocele.

### 3.4. The Pooled Prevalence of CE in Isthmocele

**Figure 3:**
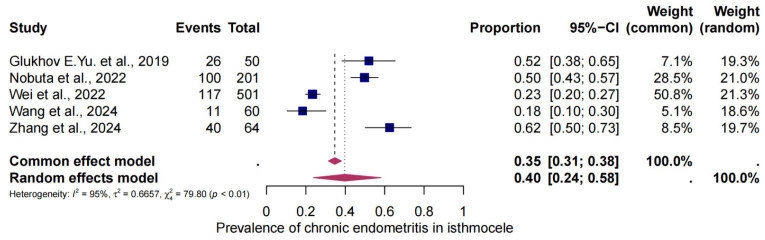
Pooled global of CE in patients with isthmoceles Pooled prevalence of CE in patients with isthmoceles [25,26,27,28,31]. Forest plot of proportions and 95% confidence intervals (CIs) for studies evaluating the prevalence of CE in patients with isthmocele. The blue squares for each study indicate the proportion; the size of the boxes indicates the weight of the study; and the horizontal lines indicate the 95% CI. Data in bold and pink diamonds represent the pooled prevalence for CE and 95% CI. Overall estimates are shown in the fixed and random-effects models.

Five studies were eligible for analysis of the prevalence of CE in patients with an isthmocele. The overall prevalence was 40% (95% CI: 24–58%). The test for heterogeneity showed significant heterogeneity between the studies (I^2^ = 95, *p* < 0.01) (Figure 3).

### 3.5. The Risk of CE in Isthmocele

**Figure 4:**
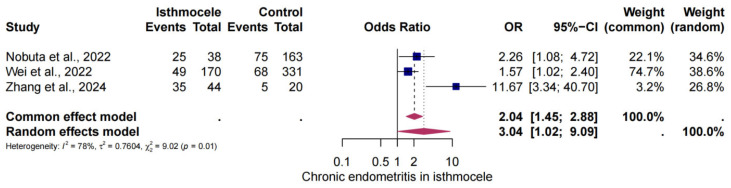
The risk of CE in isthmocele [25,26,31]. Forest plot of proportions and 95% confidence intervals (CIs) for studies evaluating the risk of CE in patients with and without isthmocele. Overall estimates are shown in the fixed and random-effects models.

A total of three studies were selected for analysis in order to determine the risk of CE in women patients with an isthmocele compared to women without an isthmocele. The analysis yielded a threefold elevated risk of CE in patients with an isthmocele compared to those without. The presence of an isthmocele was found to be significantly associated with a higher risk of CE than its absence (OR = 3.01; 95% CI: 1.02–9.03). The heterogeneity observed in this analysis was I^2^ = 78%, *p* < 0.01 (Figure 4).

### 3.6. The Risk of CE in AUB and Isthmocele

**Figure 5:**
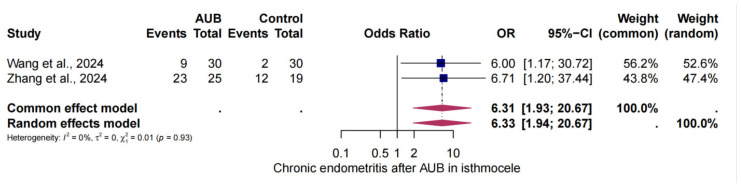
The risk of CE in AUB and isthmocele [26,27]. Forest plot of proportions and 95% confidence intervals (CIs) for studies evaluating the risk of CE in AUB in patients with and without isthmocele. Overall estimates are shown in the fixed and random-effects models.

Two studies were selected to analyse the risk of CE in women with AUB and isthmocele compared [29,30] to women with AUB without isthmocele. The analysis demonstrated that the probability of CE in the presence of AUB was found to be six-fold higher than in the absence of AUB and isthmocele (OR = 6.33, 95% CI: 1.94–20.67). Heterogeneity was observed in this analysis (I^2^ = 0%, *p* < 0.01) (Figure 5).

## 4. Discussion

The aim of this systematic review and meta-analysis was to evaluate the association between isthmocele and CE. To our knowledge, this is the first meta-analysis to assess the pooled prevalence of CE in isthmocele and the risk for CE in women with and without isthmocele.

Our study revealed the following important findings: First, the overall pooled prevalence of CE in isthmocele is approximately 40%. Second, the prevalence of CE in the presence of isthmocele is approximately three times higher than that in those without isthmocele (OR = 3.01; 95% CI: 1.02–9.03). Finally, the prevalence of CE is even higher in women with isthmocele-associated AUB (OR = 6.33, 95% CI: 1.94–20.67).

Despite the high clinical relevance of isthmocele and CE, the quality of studies on this topic is poor. There were three studies of good quality [23,24,25], three of poor quality [26,27,28], and one of fair quality [30]. Including a control group is essential for an objective assessment of the association studied.

Our results support a strong association between CE and isthmocele. By infiltrating the endometrial stroma, plasma cells secrete cytokines that are involved in the immune response during wound healing [35]. Consequently, an unbalanced immune response can lead to chronic inflammation, which may contribute to the formation of an isthmocele [16,30]. This hypothesis is further substantiated by the observation of an increase in the expression of inflammatory cytokines (Figure 2), which has led to its classification as a chronic disorder. This is evidenced by the study by Zhang et al. in 2024, who found CE in 79.6% of patients with isthmocele, but only in 25% of women without isthmocele [30]. These findings are consistent with those reported by Wei et al., 2022 [28] who observed an association between CE and isthmocele in patients with infertility. The results obtained in this study demonstrate the high prevalence of CE and the association with isthmocele (OR = 3.01; 95% CI: 1.02–9.03).

The major clinical symptom of isthmocele is considered to be abnormal menstrual and uterine bleeding. One hypothesis suggests that menstrual blood is retained and secreted intermittently, as a result of alterations in the contractility of the uterine musculature [13,14] or extravasation of blood from fragile vessels [15,36]. Morris et al. hypothesises that adenomyosis or endometriosis in the niche could lead to local blood production and consequently fluid accumulation [37]. Inflammation and capillary structural integrity have been identified in numerous scientific studies as factors contributing to the development of erosions and bleeding [38]. Our results confirm that individuals with AUB have a significantly increased risk of CE (OR = 6.33, 95% CI: 1.94–20.67).

Endometriosis is defined as a pathological condition characterised by the presence of endometrial tissue in locations other than those typically associated with its normal location [39]. However, endometriosis is often accompanied by an isthmocele. Endometriosis can be a cause of infertility due to chronic inflammation and anatomical changes caused by adhesions [40]. A retrospective study by Gulz et al. found that 27% of patients with isthmocele who underwent laparoscopic resection had endometriosis [40]. Conversely, Nobuta et al. observed a higher incidence of endometriosis in patients with infertility associated with isthmocele (70%) [34].

Endometrial microbial disease, a topic of importance in the field of reproductive health research, has seen a significant increase in research activity in recent years [22,41]. This condition is typified by a considerable imbalance in the composition of the microbiome within the female reproductive system, accompanied by persistent inflammation [42]. Hsu et al. [43] reported bacterial colonisation of the isthmocele in women with secondary infertility. Yang et al. [33] found that alteration of the uterine microbiota in patients with isthmocele may be closely related to local inflammation, reinforcing the established association between CE and isthmocele. Inflammatory cytokines and their role in endometrial stromal lesions have been studied in relation to the process of chronic uterine inflammation [17,21]. Tortorella et al. and Nobuta et al. demonstrated that levels of TNF-α and IL-1β are elevated in women with isthmocele [34,44].

The prevalence of these conditions and their impact on reproduction have become increasingly prominent subjects of research in recent decades. The absence of a standardised definition and diagnostic criteria for CE complicates research in this field. CE is daignosed by immunohistochemical staining of the CD138 cell marker, which is more sensitive and accurate than hematoxylin-eosin staining. No consensus exists on the specific plasma cell threshold required to diagnose the disease [41,45].

One of the aspects that has been most extensively debated is the approach to CE. The primary treatment modality is oral antibiotics, with doxycycline being the most prevalent first-line regimen, administered at a dose of 100 mg twice daily for a duration of 14 days [23,46]. Combinations of medications have been utilised within the domain of alternative medicine, including the administration of ciprofloxacin 500 mg once or twice daily, in conjunction with metronidazole 500 mg once a day for a duration of two weeks [47]. However, in cases where resistance has been identified or where specific microbiological findings have been documented, targeted regimens have been prescribed. In cases of recurrent implantation failure and resistance to CE, intrauterine infusion of antibiotics, guided by an antibiogram, has been successfully trialled, achieving clinical pregnancies in a number of cases [48]. The therapeutic regimen should be personalised, taking into account aetiological factors and clinical contexts, particularly in women with reproductive dysfunction or undergoing assisted reproductive procedures. However, it is imperative to consider that the persistence rate of CE after three cycles of oral antibiotics is estimated at approximately 25%. Consequently, further reflection is required on the potential for antibiotic resistance in the future [48].

The optimisation of diagnostic criteria and validation of treatment for CE in terms of clinical outcomes, preferably in the context of randomised trials, are recommended [49]. Given the prevalence and severity of CE, future randomised controlled trials are needed. Future investigations should focus on elucidating the precise pathophysiological mechanisms driving the development of CE in the presence of isthmocele. This includes exploring the role of inflammation, altered uterine hemodynamics, and the impact of specific surgical techniques. Additionally, the development of targeted interventions, including optimised surgical approaches and pharmacological therapies, is essential for the prevention and effective management of CE. Ultimately, improving our understanding of this complex relationship will lead to enhanced patient outcomes and a reduction in the morbidity associated with caesarean scar defects.

Our findings emphasise the necessity for public health initiatives to reduce the prevalence of unnecessary caesarean sections, given the well-documented long-term risks associated with the formation of isthmocele and, consequently, CE. Such conditions have the capacity to impact patients’ quality of life and compromise their future reproductive function.

Even though our study strictly followed the recommendations to provide high-quality summary reports of evidence, some limitations are evident. First, the number of the included studies was limited, and the quality of these studies was poor. Second, the definition of CE remains unclear, with CD138 predominating as the criterion. Third, the included studies did not provide detailed information on the surgical technique employed during caesarean sections, which may be a relevant factor in the development of isthmocele. Finally, a potential limitation is that none of the included studies accounted for possible confounding factors such as autoimmune diseases, which are known to be associated with increased plasma cell infiltration. This may have affected the histological interpretation of the CE.

Our meta-analysis demonstrates a significant association between isthmocele and CE. However, the quality and selection of control groups in the included studies represent a relevant methodological limitation. In accordance with the majority of the existing literature in this field, many of the studies have small samples, retrospective designs, and heterogeneous diagnostic criteria, which may impact the robustness of the results. In this context, this paper calls for the urgent necessity of conducting randomised clinical trials with well-defined and clinically comparable control groups, in order to facilitate the establishment of more robust conclusions. Our meta-analysis, therefore, not only provides preliminary evidence relevant to the topic, but also emphasises the importance of improving methodological design in future research on this important issue.

In conclusion, this systematic review and meta-analysis highlights the substantial clinical burden imposed by the high prevalence of CE in women with isthmocele, revealing that approximately 40% of this population experiences this condition. Notably, our findings demonstrate a compelling association between isthmocele and CE. Our results underscore the critical importance of recognising isthmocele as a potential risk factor for CE, particularly in women with isthmocele-associated AUB. This recognition necessitates a heightened clinical awareness to facilitate earlier detection and more effective management strategies. Well-designed prospective randomised controlled trials are essential to accurately assess the efficacy of antibiotic therapy in patients with isthmocele, considering the limited availability and poor quality of the current data.

## Data Availability

This study was based on the results of relevant published studies.

## References

[1] Tower A.M., Frishman G.N. (2013). Cesarean Scar Defects: An Underrecognized Cause of Abnormal Uterine Bleeding and Other Gynecologic Complications. J. Minim. Invasive Gynecol..

[2] Bij de Vaate A.J.M., Brölmann H.A.M., van der Voet L.F., van der Slikke J.W., Veersema S., Huirne J.A.F. (2011). Ultrasound Evaluation of the Cesarean Scar: Relation between a Niche and Postmenstrual Spotting. Ultrasound Obstet. Gynecol..

[3] van der Voet L.F., Bij de Vaate A.M., Veersema S., Brölmann H.A.M., Huirne J.A.F. (2014). Long-Term Complications of Caesarean Section. The Niche in the Scar: A Prospective Cohort Study on Niche Prevalence and Its Relation to Abnormal Uterine Bleeding. BJOG.

[4] Tulandi T., Cohen A. (2016). Emerging Manifestations of Cesarean Scar Defect in Reproductive-Aged Women. J. Minim. Invasive Gynecol..

[5] Betran A.P., Ye J., Moller A.-B., Souza J.P., Zhang J. (2021). Trends and Projections of Caesarean Section Rates: Global and Regional Estimates. BMJ Glob. Health.

[6] Florio P., Filippeschi M., Moncini I., Marra E., Franchini M., Gubbini G. (2012). Hysteroscopic Treatment of the Cesarean-Induced Isthmocele in Restoring Infertility. Curr. Opin. Obstet. Gynecol..

[7] Vachon-Marceau C., Demers S., Bujold E., Roberge S., Gauthier R.J., Pasquier J.-C., Girard M., Chaillet N., Boulvain M., Jastrow N. (2017). Single versus Double-Layer Uterine Closure at Cesarean: Impact on Lower Uterine Segment Thickness at next Pregnancy. Am. J. Obstet. Gynecol..

[8] Di Spiezio Sardo A., Saccone G., McCurdy R., Bujold E., Bifulco G., Berghella V. (2017). Risk of Cesarean Scar Defect Following Single- vs. Double-layer Uterine Closure: Systematic Review and Meta-analysis of Randomized Controlled Trials. Ultrasound Obstet. Gynecol..

[9] Alper E., Aksakal E., Usta I., Urman B. (2024). The Novel Parallel Closure Technique Compared to Single-Layer Closure of the Uterus After Primary Cesarean Section Decreases the Incidence of Isthmocele Formation and Increases Residual Myometrial Thickness. Cureus.

[10] Asoglu M.R., Celik C., Ozturk E., Cavkaytar S., Bahceci M. (2021). Impact of Isthmocele on Assisted Reproductive Treatment Outcomes: An Age-Matched Retrospective Study. J. Minim. Invasive Gynecol..

[11] Vitagliano A., Cicinelli E., Viganò P., Sorgente G., Nicolì P., Busnelli A., Dellino M., Damiani G.R., Gerli S., Favilli A. (2024). Isthmocele, Not Cesarean Section per Se, Reduces in Vitro Fertilization Success: A Systematic Review and Meta-Analysis of over 10,000 Embryo Transfer Cycles. Fertil. Steril..

[12] Vidal A., Geiger J., Vinayahalingam V., Pape J., Gulz M., Karrer T., Mueller M.D., Von Wolff M. (2025). High Live Birth Rates after Laparoscopic Isthmocele Repair in Infertility: A Systematic Review and Meta-Analysis. Front. Endocrinol..

[13] Vidal A., Bora C., Von Holzen J., Gulz M., Obmann V.C., Pape J., Karrer T., Yilmaz G., Von Wolff M. (2025). Cine-MRI for Quantifying Uterine Peristalsis: A Systematic Review and Meta-Analysis. J. Clin. Med..

[14] Vidal A., Trejos V., Pape J., Karrer T., Yilmaz G., Von Wolff M. (2025). Lower Pregnancy Rate in Women with High Uterine Peristalsis before Embryo Transfer: A Systematic Review and Meta-Analysis. Reprod. Biol. Endocrinol..

[15] Vissers J., Hehenkamp W., Lambalk C.B., Huirne J.A. (2020). Post-Caesarean Section Niche-Related Impaired Fertility: Hypothetical Mechanisms. Hum. Reprod..

[16] Baldini G.M., Lot D., Malvasi A., Di Nanni D., Laganà A.S., Angelucci C., Tinelli A., Baldini D., Trojano G. (2024). Isthmocele and Infertility. J. Clin. Med..

[17] Greenwood S.M., Moran J.J. (1981). Chronic Endometritis: Morphologic and Clinical Observations. Obstet. Gynecol..

[18] Bouet P.-E., El Hachem H., Monceau E., Gariépy G., Kadoch I.-J., Sylvestre C. (2016). Chronic Endometritis in Women with Recurrent Pregnancy Loss and Recurrent Implantation Failure: Prevalence and Role of Office Hysteroscopy and Immunohistochemistry in Diagnosis. Fertil. Steril..

[19] Kimura F., Takebayashi A., Ishida M., Nakamura A., Kitazawa J., Morimune A., Hirata K., Takahashi A., Tsuji S., Takashima A. (2019). Review: Chronic Endometritis and Its Effect on Reproduction. J. Obstet. Gynaecol..

[20] Moreno O.M., Paredes A.C., Suarez-Obando F., Rojas A. (2021). An Update on Fanconi Anemia: Clinical, Cytogenetic and Molecular Approaches (Review). Biomed. Rep..

[21] Kitaya K., Takeuchi T., Mizuta S., Matsubayashi H., Ishikawa T. (2018). Endometritis: New Time, New Concepts. Fertil. Steril..

[22] Cicinelli E., Matteo M., Trojano G., Mitola P.C., Tinelli R., Vitagliano A., Crupano F.M., Lepera A., Miragliotta G., Resta L. (2018). Chronic Endometritis in Patients with Unexplained Infertility: Prevalence and Effects of Antibiotic Treatment on Spontaneous Conception. Am. J. Rep. Immunol..

[23] Cicinelli E., Matteo M., Tinelli R., Lepera A., Alfonso R., Indraccolo U., Marrocchella S., Greco P., Resta L. (2015). Prevalence of Chronic Endometritis in Repeated Unexplained Implantation Failure and the IVF Success Rate after Antibiotic Therapy. Hum. Reprod..

[24] Page M.J., McKenzie J.E., Bossuyt P.M., Boutron I., Hoffmann T.C., Mulrow C.D., Shamseer L., Tetzlaff J.M., Akl E.A., Brennan S.E. (2021). The PRISMA 2020 Statement: An Updated Guideline for Reporting Systematic Reviews. BMJ.

[25] Borissov N., Haas Q., Minder B., Kopp-Heim D., von Gernler M., Janka H., Teodoro D., Amini P. (2022). Reducing Systematic Review Burden Using Deduklick: A Novel, Automated, Reliable, and Explainable Deduplication Algorithm to Foster Medical Research. Syst. Rev..

[26] Van der Mierden S., Tsaioun K., Bleich A., Leenaars C.H.C. (2019). Software Tools for Literature Screening in Systematic Reviews in Biomedical Research. ALTEX.

[27] Wells G.A., Shea B., O’Connell D., Peterson J., Welch V., Losos M., Tugwell P. (2009). The Newcastle-Ottawa Scale (NOS) for Assessing the Quality of Nonrandomised Studies in Meta-Analyses.

[28] Wei L., Xu C., Zhao Y., Zhang C. (2022). Higher Prevalence of Chronic Endometritis in Women with Cesarean Scar Defect: A Retrospective Study Using Propensity Score Matching. J. Pers. Med..

[29] Wang Y., Han Y., Guo X., Wei Q., Xia Y., Gao L., Wang H., Lu X., Shu J. (2025). Caesarean Scar Endometrial Defects Contribute to Post-Caesarean Abnormal Uterine Bleeding and Chronic Endometritis: A Retrospective Case–Control Study. BJOG.

[30] Zhang J., Huang J., Xu Z., Yang Q., Zeng L., Zhou L., Deng K. (2024). The Correlation between Chronic Endometritis and Caesarean Scar Diverticulum. J. Reprod. Immunol..

[31] Glukhov E.Y., Dikke G.B., Neff E.I., Glukhova V.E., Svyazhina A.V. (2019). Chronic Endometritis and an Incompetent Uterine Scar after Cesarean Section: Long-Term Outcomes of Metroplasty. Obstet. Gynecol..

[32] Higuchi A., Tsuji S., Nobuta Y., Nakamura A., Katsura D., Amano T., Kimura F., Tanimura S., Murakami T. (2022). Histopathological Evaluation of Cesarean Scar Defect in Women with Cesarean Scar Syndrome. Reprod. Med. Biol..

[33] Yang X., Pan X., Cai M., Zhang B., Liang X., Liu G. (2021). Microbial Flora Changes in Cesarean Section Uterus and Its Possible Correlation with Inflammation. Front. Med..

[34] Nobuta Y., Tsuji S., Kitazawa J., Hanada T., Nakamura A., Zen R., Amano T., Murakami T. (2022). Decreased Fertility in Women with Cesarean Scar Syndrome Is Associated with Chronic Inflammation in the Uterine Cavity. Tohoku J. Exp. Med..

[35] Puente E., Alonso L., Laganà A.S., Ghezzi F., Casarin J., Carugno J. (2020). Chronic Endometritis: Old Problem, Novel Insights and Future Challenges. Int. J. Fertil. Steril..

[36] Donnez O., Donnez J., Orellana R., Dolmans M.-M. (2017). Gynecological and Obstetrical Outcomes after Laparoscopic Repair of a Cesarean Scar Defect in a Series of 38 Women. Fertil. Steril..

[37] Morris H. (1995). Surgical Pathology of the Lower Uterine Segment Caesarean Section Scar: Is the Scar a Source of Clinical Symptoms?. Int. J. Gynecol. Pathol..

[38] Dominguez J.A., Pacheco L.A., Moratalla E., Carugno J.A., Carrera M., Perez-Milan F., Caballero M., Alcázar J.L. (2023). Diagnosis and Management of Isthmocele (Cesarean Scar Defect): A SWOT Analysis. Ultrasound Obstet. Gynecol..

[39] Becker C.M., Bokor A., Heikinheimo O., Horne A., Jansen F., Kiesel L., King K., Kvaskoff M., Nap A., Petersen K. (2022). ESHRE Guideline: Endometriosis. Hum. Reprod. Open.

[40] Gulz M., Imboden S., Nirgianakis K., Siegenthaler F., Rau T.T., Mueller M.D. (2022). Endometriosis and Isthmocele: Common or Rare?. J. Clin. Med..

[41] Cicinelli E., Matteo M., Tinelli R., Pinto V., Marinaccio M., Indraccolo U., De Ziegler D., Resta L. (2014). Chronic Endometritis Due to Common Bacteria Is Prevalent in Women with Recurrent Miscarriage as Confirmed by Improved Pregnancy Outcome After Antibiotic Treatment. Reprod. Sci..

[42] Lozano F.M., Bernabeu A., Lledo B., Morales R., Diaz M., Aranda F.I., Llacer J., Bernabeu R. (2021). Characterization of the Vaginal and Endometrial Microbiome in Patients with Chronic Endometritis. Eur. J. Obstet. Gynecol. Reprod. Biol..

[43] Hsu I., Hsu L., Dorjee S., Hsu C.-C. (2022). Bacterial Colonization at Caesarean Section Defects in Women of Secondary Infertility: An Observational Study. BMC Pregnancy Childbirth.

[44] Tortorella C., Piazzolla G., Matteo M., Pinto V., Tinelli R., Sabbà C., Fanelli M., Cicinelli E. (2014). Interleukin-6, Interleukin-1β, and Tumor Necrosis Factor α in Menstrual Effluents as Biomarkers of Chronic Endometritis. Fertil. Steril..

[45] Song D., Feng X., Zhang Q., Xia E., Xiao Y., Xie W., Li T.C. (2018). Prevalence and Confounders of Chronic Endometritis in Premenopausal Women with Abnormal Bleeding or Reproductive Failure. Reprod. Biomed. Online.

[46] Cheng X., Huang Z., Xiao Z., Bai Y. (2022). Does Antibiotic Therapy for Chronic Endometritis Improve Clinical Outcomes of Patients with Recurrent Implantation Failure in Subsequent IVF Cycles? A Systematic Review and Meta-Analysis. J. Assist. Reprod. Genet..

[47] Liu J., Liu Z.A., Liu Y., Cheng L., Yan L. (2022). Impact of Antibiotic Treatment for Chronic Endometritis on Pregnancy Outcomes in Women with Reproductive Failures (RIF and RPL): A Systematic Review and Meta-Analysis. Front. Med..

[48] Di Gennaro F., Guido G., Frallonardo L., Pennazzi L., Bevilacqua M., Locantore P., Vitagliano A., Saracino A., Cicinelli E. (2025). Chronic Endometritis and Antimicrobial Resistance: Towards a Multidrug-Resistant Endometritis? An Expert Opinion. Microorganisms.

[49] Strug M.R., Hartup L.A., Ryan E., Lathi R.B. (2024). Unveiling the Silver Lining: A Narrative Review of Clinical Evaluation and Management of Chronic Endometritis and Its Impact on Fertility. FS Rev..

